# Supplementary material for: The Impact of Consuming Zinc-Biofortified Wheat Flour on Haematological Indices of Zinc and Iron Status in Adolescent Girls in Rural Pakistan: A Cluster-Randomised, Double-Blind, Controlled Effectiveness Trial
Source: Nutrients. 2022 Apr 15;14(8):1657. doi: 10.3390/nu14081657 (PMC9026921; doi:10.3390/nu14081657)
Supplement: Supplementary file 1 [file nutrients-14-01657-s001.zip › Suppl_TableS1_primary MS_FV.pdf]

Table S1. Spearman correlation coefficient between plasma zinc concentration and inflammatory markers.

		**Plasma Zinc**	**C-Reactive Protein**	**Alpha-1-Acid Glycoprotein**
Plasma Zinc	r_s	1.000	-0.027	-0.020
	N	2177	2131	2170
C-Reactive Protein	r_s	0.027	1.000	0.456*
	N	2131	2154	2150
Alpha-1-Acid	r_s	-0.020	0.456*	1.000
Glycoprotein	N	2170	2150	2193

*Correlation significant at the p = 0.01 level.
r_s, Spearman's rho correlation coefficient.
