# Supplementary material for: The Impact of Consuming Zinc-Biofortified Wheat Flour on Haematological Indices of Zinc and Iron Status in Adolescent Girls in Rural Pakistan: A Cluster-Randomised, Double-Blind, Controlled Effectiveness Trial
Source: Nutrients. 2022 Apr 15;14(8):1657. doi: 10.3390/nu14081657 (PMC9026921; doi:10.3390/nu14081657)
Supplement: Supplementary file 1 [file nutrients-14-01657-s001.zip › Suppl_TableS2_primary MS_FV.pdf]

Table S2. Haemoglobin and other haematological measures by study arms at baseline, mid-point and endline.

Outcomes	Time Points	n	Control	n	Intervention	β (95%CI)*	X^2	t	p
	Baseline	219	12.9 ± 1.3	200	12.8 ± 1.1				
Haemoglobin, Hb (g/dL)	Midpoint	214	12.8 ± 1.6	192	13.0 ± 1.5	0.232 (-0.054, 0.518)		1.640	0.109
	Edline	213	12.9 ± 1.4	186	12.9 ± 1.4	0.002 (-0.279, 0.283)		0.013	0.990
Anaemia (Hb <11.5 g/dL	Baseline	219	39 (17.8)	200	30 (15.0)		0.599		0.439
for <12 years or <12.0	Midpoint	214	36 (16.8)	192	28 (14.6)		0.382[a]		0.536
g/dL for>=2 years)	Endline	213	36 (16.9)	186	35 (18.8)		0.249[a]		0.618
	Baseline	220	37.5 ± 3.1	200	37.5 ± 2.8				
Haematocrit, HCT (%)	Midpoint	210	38.1 ± 3.3	191	38.6 ± 3.7	0.576 (-0.214, 1.365)		1.473	0.148
	Endline	210	37.6 ±3.3	185	37.4 ± 3.4	-0.200 (-0.949, 0.550)		-0.540	0.593
	Baseline	220.0	21 (9.5)	200	12 (6.0)		1.819		0.177
HCT <33.5 %	Midpoint	210	14 (6.7)	191	13 (6.8)		0.003		0.956
	Endline	210	18 (8.6)	185	16 (8.6)		.001		0.978
Mean corpuscular	Baseline	220	34.2 ± 1.3	200	34.1 ± 1.3				
haemoglobin	Midpoint	213	33.8 ± 1.3	193	33.8 ± 1.2	0.038 (-0.250, 0.326)		0.267	0.791
concentration, MCHC (g/dL)	Endline	212	34.4 ± 1.5	188	34.7 ± 1.3	0.338 (-0.097, 0.773)		1.564	0.125
	Baseline	220.0	18 (8.2)	200	16 (8.0)		0.005		0.946
MCHC <32.3 g/dL	Midpoint	213	26 (12.2)	193	22 (11.4)		0.063		0.801
	Endline	212	20 (9.4)	188	9 (4.8)		3.199		0.074
	Baseline	217	82.0 ± 7.5	200	82.3 ± 6.4				
Mean corpuscular volume, MCV (fL)	Midpoint	213	82.9 ± 7.6	191	83.1 ± 6.8	0.107 (-0.958, 1.171)		0.205	0.839
	Endline	213	83.4 ± 8.1	188	83.1 ± 7.0	-0.883 (-2.283, 0.517)		-1.287	0.208
	Baseline	217	23 (10.6)	200	18 (9.0)		0.300	0.584	
MCV <74.7 fL	Midpoint	213	22 (10.3)	191	16 (8.4)		0.450	0.502	
	Endline	213	23 (10.8)	188	15 (8.0)		0.925	0.336	
	Baseline	220	4.6 ± 0.5	200	4.6 ± 0.5				
Red blood cell count, RBC count (10^6/μL)	Midpoint	210	4.6 ± 0.5	191	4.7 ± 0.5	0.073 (-0.023, 0.168)		1.541	0.133
	Endline	213	4.5 ± 0.5	187	4.5 ± 0.5	0.020 (-0.080, 0.120)		0.400	0.691
	Baseline	220	7 (3.2)	200	9 (4.5%)		0.497		0.481
RBC count <3.84X10^6 /μL	Midpoint	210	8 (3.8)	191	7 (3.7%)		0.006		0.939
	Endline	213	13 (6.1)	187	16 (8.6%)		0.891		0.345

Data presented as Mean ± SD or n (%).

*Values represent beta coefficient and 95% CI from linear regression models.

p values obtained using linear mixed models adjusted for cluster effect and baseline values to test differences between the groups for continuous variable. Categorical variables by Pearson's chi-square test. Significant was set at $p<0.05$.
