# Supplementary material for: The Impact of Consuming Zinc-Biofortified Wheat Flour on Haematological Indices of Zinc and Iron Status in Adolescent Girls in Rural Pakistan: A Cluster-Randomised, Double-Blind, Controlled Effectiveness Trial
Source: Nutrients. 2022 Apr 15;14(8):1657. doi: 10.3390/nu14081657 (PMC9026921; doi:10.3390/nu14081657)
Supplement: Supplementary file 1 [file nutrients-14-01657-s001.zip › Suppl_TableS3__primary MS_FV.pdf]

Table S3. Prevalence of various grades of anaemia by study arms at baseline, midpoint and endline.

Time Points	Anaemia Garde *	n	Control	n	Intervention	X^2	P
Baseline	Mild	219	23 (10.5)	200	20 (10.0)	1.637	0.651
	Moderate		15 (6.8)		10 (5.0)		
	Severe		1(0.5)		0 (0.0)		
	None		180 (82.2)		170 (85.0)		
Midpoint	Mild	214	20 (9.3)	192	16 (8.3)	1.162	0.762
	Moderate		11 (5.1)		10 (5.2)		
	Severe		5 (2.3)		2 (1.0)		
	None		178 (83.2)		164 (85.4)		
Endline	Mild	213	19 (8.9)	186	21 (11.3)	0.691	0.875
	Moderate		14 (6.6)		12 (6.5)		
	Severe		3 (1.4)		2 (1.1)		
	None		177 (83.1)		151 (81.2)		

Data presented as n (%).
*Mild (Hb=11.0-11.4 g/dL for <12 years or 11.0-11.9 g/dL for >=12 years); Moderate (Hb=8.0-10.9 g/dL); Severe (Hb <8.0 g/dL); Non-anaemic (Hb >=11.5 g/dL for <12 years or >=12.0 g/dL for >=12 years)
P values obtained by Pearson's chi-square test. Significance was set at p<0.05.
