# Supplementary figures and images for: The Impact of Consuming Zinc-Biofortified Wheat Flour on Haematological Indices of Zinc and Iron Status in Adolescent Girls in Rural Pakistan: A Cluster-Randomised, Double-Blind, Controlled Effectiveness Trial
Source: Nutrients. 2022 Apr 15;14(8):1657. doi: 10.3390/nu14081657 (PMC9026921; doi:10.3390/nu14081657)

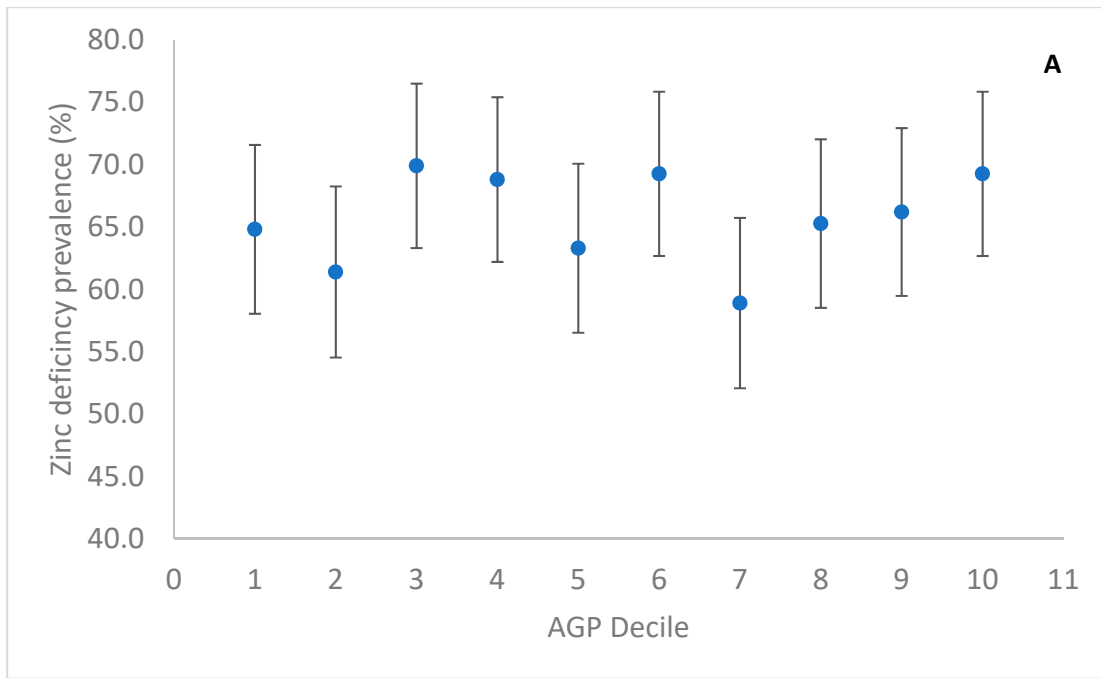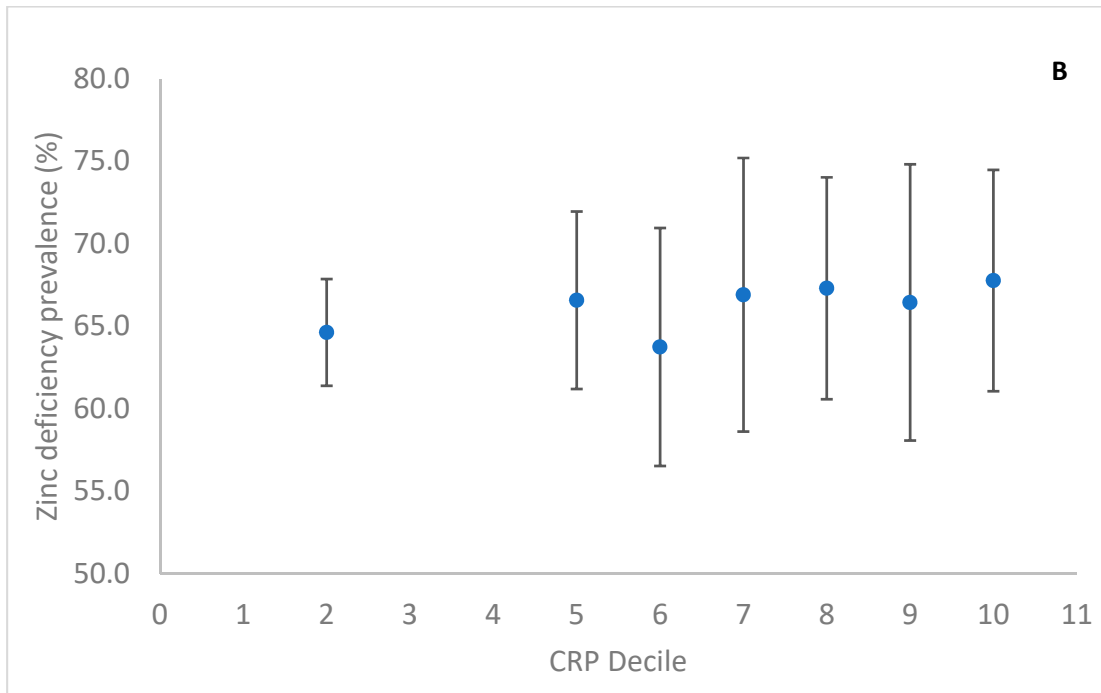

Supplement: Supplementary file 1 [file nutrients-14-01657-s001.zip › Suppl_FigureS1__primary MS_FV.pdf]
